# Clincopathological characteristics and treatment outcomes of oral leukoplakia by carbon dioxide laser excision in the elderly patients

**DOI:** 10.1002/hed.26074

**Published:** 2020-01-13

**Authors:** Shih‐Wei Yang, Yun‐Shien Lee, Liang‐Che Chang, Cheng‐Cheng Hwang, Tai‐An Chen

**Affiliations:** ^1^ Department of Otolaryngology‐Head and Neck Surgery Chang Gung Memorial Hospital Keelung Taiwan, ROC; ^2^ College of Medicine Chang Gung University Taoyuan Taiwan, ROC; ^3^ Genomic Medicine Research Core Laboratory Chang Gung Memorial Hospital Tao‐Yuan Taiwan, ROC; ^4^ Department of Biotechnology Ming Chuan University Tao‐Yuan Taiwan, ROC; ^5^ Department of Pathology Chang Gung Memorial Hospital Keelung Taiwan, ROC

**Keywords:** elderly, laser, malignant transformation, oral leukoplakia, recurrence

## Abstract

**Background:**

Older age is one of the factors associated with malignant transformation of oral leukoplakia (OL). The purpose of this study is to analyze the clincopathological features and treatment outcomes of OL in the elderly patients.

**Methods:**

The demographic data and histopathological results of the patients (age ≥ 65) who received carbon dioxide laser surgery for OL from 2002 to 2017 were analyzed statistically.

**Results:**

There were 53 males and 16 females, with a mean age 71.2 ± 4.9. The follow‐up time was 42.5 ± 35.2 months. In the univariate analysis, morphology, pathology, and area were found to be factors associated with postoperative recurrence. Among these factors, pathology and area were the independent predictive factors for recurrence in the multivariate logistic regression model. Malignant transformation occurred in 8 of 69 patients (11.6%).

**Conclusions:**

The pathological high‐risk dysplasia and area of OL were the two prognostic factors for postoperative recurrence.

## 1 INTRODUCTION

Oral cancers are well known for their functional destruction and cosmetic disfiguration after radical surgery and/or chemoradiation. Postoperatively rehabilitation programs, including speech therapy, swallow training, and physical rehabilitation, are usually needed for patients with oral cancers. Oral potentially malignant disorders (OPMDs) are conditions that precede the occurrence of oral squamous cell carcinoma,^1^, ^2^, ^3^ which are lesions characteristically confined to the mucosa without invasion. An early intervention to remove the OPMD lesions is often not as destructive as radical surgeries for oral cancers. A range of oral mucosa diseases with an elevated risk of malignant transformation has been described and studied.^1^, ^2^, ^4^ These diseases include leukoplakia, erythroplakia, oral lichen planus, oral submucous fibrosis, actinic cheilitis, palatal lesions of reverse cigar smoking, discoid lupus erythematosus, and some inherited disorders, such as dyskeratosis congenita and Fanconi anemia.^1^, ^2^, ^4^ Among OPMDs, oral leukoplakia (OL) is the most commonly encountered type.^1^, ^3^, ^4^, ^5^, ^6^ The factors associated with malignant transformation of OL are also extensively studied, including grade of dysplasia, advanced age, female sex, lesion area exceeding 200 mm^2^, and the nonhomogeneous type.^1^, ^6^, ^7^, ^8^, ^9^, ^10^, ^11^, ^12^, ^13^, ^14^ A general pattern of increased risk of malignant transformation with advanced age was observed in a systematic review of observational studies on OL.^8^ In a retrospective study of 320 patients with OL, people of older age (>60 years) had higher risk of malignant transformation.^11^ In another retrospective study of 167 patients, age of operated patients was one of the factors significantly predictive of the development of recurrence, new lesions, and malignant transformation of OL.^14^ Elderly patients with OL seem to be a group at higher risk of malignant transformation. Older adults were more affected in the domains of “physical and functional limitation” and “effect of treatment in daily life” in a study on the quality of life of patients with OL.^15^ Besides, old patients may have more difficulties coping with the treatment modalities due to their general condition and comorbidities; regular commutes to appointments are also a challenge to them.^15^ There are few studies in the literature addressing the characteristics of OL in the elderly patients. The aim of this study was to analyze the clincopathological features and evaluate the treatment outcomes of OL in the elderly patients.

## 2 MATERIALS AND METHODS

This study has been approved by the Institutional Review Board of Chang Gung Memorial Hospital (certificate number: 201901384B0). Patients with OL who received transoral laser excision at the Department of Otolaryngology‐Head and Neck Surgery of Keelung Chang Gung Memorial Hospital, from July 2002 to September 2017, were enrolled and all medical charts were retrospectively reviewed.

Preoperatively, all patients received thorough oral cavity examination at the outpatient department. Written informed consent was signed by every patient before surgical intervention. Some of the patients received carbon dioxide (CO_2_) laser surgery after an incisional biopsy for OL while some underwent laser excision directly. The procedure was performed as previously described.^16^, ^17^, ^18^ All the OL lesions were excised and the specimens were sent for permanent pathological examination. No laser vaporization was performed. Before surgery, all the OL lesions were photographed and the types of OL were initially assessed by the author (S.‐W.Y.) and the photographs were reviewed by two otolaryngologists, including homogeneous and nonhomogenous types.^19^, ^20^, ^21^, ^22^ The subtypes of nonhomogeneous OL, including speckled, nodular, and verruciform types, were also recorded.^1^, ^4^, ^20^ Inclusion criteria consisted of a clinical diagnosis of OL in the oral cavity mucosa, and patients' age older than 65 years. Patients with an initial pathological diagnosis of invasive carcinoma, aged younger than 65 years, clinical diagnoses other than OL (such as erythroplakia, lichen planus, submucous fibrosis etc.), and postoperative follow‐up time less than 3 months, were excluded. Other exclusion criteria were previous treatments of OL at other hospitals, oropharyngeal lesions, and pathological diagnosis of verruciform hyperplasia. The history of betel quid chewing, alcohol drinking, and tobacco use were obtained by detailed questioning at the patients' first visit to the clinic; criteria for a positive assignment were defined as previously mentioned.^17^ Demographic and clinicopathological data, information on malignant transformation into carcinoma, and recurrence of OL after surgical treatment were reviewed retrospectively. All the patients recruited in this study were newly diagnosed and received surgical excision of OL lesion(s) with CO_2_ laser. Hematoxylin and eosin‐stained (H&E) slides were examined by two independent pathologists to confirm the diagnoses and determine the degree of epithelial dysplasia and existence of invasive carcinoma. An electronic medical chart software system, installed in 2004, allowed all chart records to be thoroughly reviewed.

Recurrence Recurrence was defined here as when a patient had OL recurrence at the same site, but not from the site of OL that later transformed to carcinoma if the patient had presented with multi‐focal disease. In patients with multiple sites of OL, the clinical forms and pathology of every lesion were recorded and calculated for inclusion in the statistical analysis. Due to the multiple sites, the total number of lesions and pathological results were more than the total number of the patients. Multifocal disease can be classified as synchronous or metachronous. In this study, only synchronous lesions were recorded. The area of OL in a patient was the summation of all OL lesions if a patient had more than one lesion. If a patient had recurrence, the area of recurrent lesion(s) was deducted from the total area used for the statistical calculation. If the statistical operation was calculated on a per capita basis, the most severe form of morphology and highest degree of pathology of every patient were recorded and are shown in Tables 1 and 3. If the operation was performed on a per lesion basis, every morphology and pathology were documented and are shown in Table 2.

All the surgical procedures were performed under local anesthesia.^17^ The postoperative follow‐up was uneventful. No wound bleeding, abscess formation, or other major complications occurred. All the patients were able to come back to clinic as scheduled.

The Institutional Review Board of Chang Gung Memorial Hospital has approved this study (certificate number: 201901384B0). Due to the retrospective nature of this study, the ethical committee did not require any written informed consent and waived the need for informed consent from the enrolled patients.

## 3 STATISTICAL ANALYSIS

The results were presented descriptively, with factors related to the postoperative recurrence grouped and analyzed using Fisher's exact test, χ^2^ test, and one way analysis of variance between groups for univariate analysis. The statistical tests were performed with the MATLAB version R2015a (Mathworks Inc., Natick, MA). Probabilities of less than .05 were accepted as significant.

The association of different factors with postoperative recurrence was examined using multivariate logistic regression analysis with SPSS (version 22.0, SPSS Inc, Chicago, IL). The odds ratios (OR) of various factors were calculated by logic transformation of the probability of the development of the end point. When the 95% confidence interval (CI) of the OR of a given factor did not include 1, the value was accepted as significant (*P* < .05). To adjust for the effects of each factor, these factors were simultaneously incorporated into the regression model.

Receiver operating characteristic (ROC) curve and area under the ROC curve were measures of how well the area of OL can distinguish between postoperative recurrence and nonrecurrence. The optimal prediction of the cutoff point of the area of OL was based on the Youden Index J (= maximum [sensitivity + specificity −1]).^23^ The ROC curves were performed with SPSS and the predictions and diagnostic tests were as described by Simel et al.^24^

## 4 RESULTS

**Figure 1:**
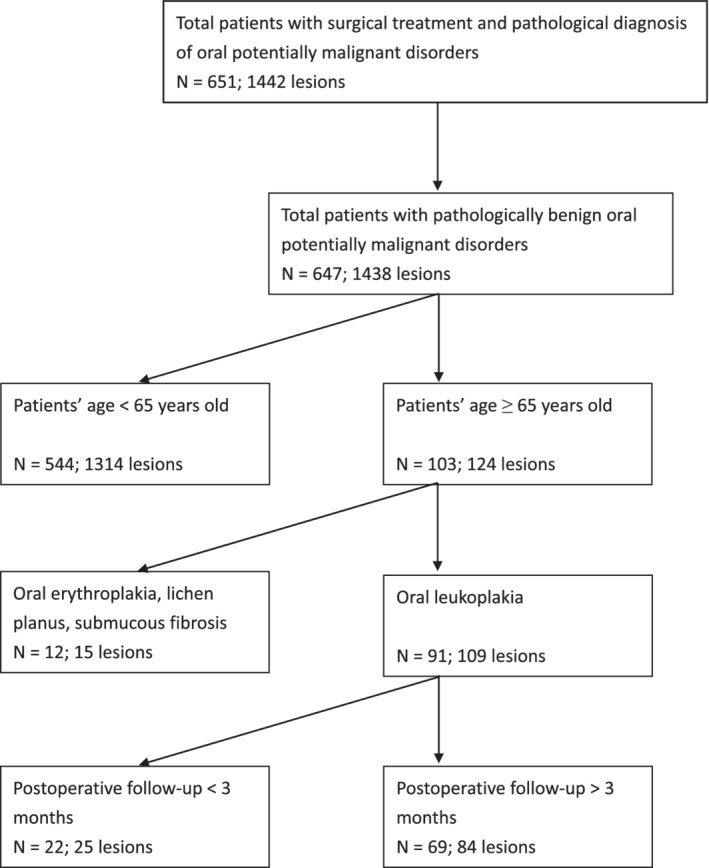
Algorithm for identifying the study cohorts

Overall, 651 patients with 1442 OPMD lesions underwent CO_2_ laser surgery at our hospital from 2002 to 2017. Excluding patients with initial diagnosis of malignant lesions, patients who were aged younger than 65 years, clinical OPMDs other than OL, and those whose postoperative follow‐up time was less than 3 months, totally 69 patients with 84 OL lesions were enrolled in this study (Figure 1). Among the 69 patients 53 were male (76.8%) and 16 were female (23.2%), whose age ranged from 65 to 83 years with a median 71.0 and average 71.2 ± 4.9. The average follow‐up time was 42.5 ± 35.2 months, median was 29.0 months. The average area of OL was 2.3 ± 1.9 cm^2^, median was 1.8 cm^2^. There were 23 patients (33.3%) who had postoperative recurrence of OL. The demographic and clinicopathological data are shown in Table 1.

**Table 1:** Clincopathological characteristics of elderly patients with oral cavity leukoplakia (n = 69)

Characteristics	Case no.	(%)
Age (y)		
Mean (SD): 71.2 (4.9)		
Median (range): 71.0 (65‐83)		
Body mass index (kg/m^2^)^a^		
Mean (SD): 24.4 (3.2)		
Gender		
Female	16	23.2
Male	53	76.8
History of head and neck cancer		
No	49	71.0
Yes	20	29.0
History of radiation therapy		
No	58	84.1
Yes	11	15.9
Cigarette smoking		
No	28	40.6
Ex‐smoker	22	31.9
Yes	19	27.5
Alcohol drinking		
No	53	76.8
Ex‐drinker	10	14.5
Yes	6	8.7
Betel quid chewing		
No	51	73.9
Ex‐chewer	18	26.1
Yes	0	0.0
Candida infection		
No	62	89.9
Yes	7	10.1
Diabetes mellitus		
No	52	75.4
Yes	17	24.6
Metformin taken		
No	58	84.1
Yes	11	15.9
Multi‐focus sites		
No	56	81.2
Yes	13	18.8
Location of leukoplakia^b^		
Buccal	54	64.3
Tongue	12	14.3
Gum	5	6.0
Labial	4	4.8
Mouth floor	1	1.2
Hard palate	1	1.2
Retromolar	7	8.3
Morphology^b^		
Homogeneous	42	50.0
Nonhomogeneous	42	50.0
Pathology		
Squamous hyperplasia	15	21.7
Mild dysplasia	34	49.3
Moderate dysplasia	12	17.4
Severe dysplasia/carcinoma in situ	8	11.6
Postoperative recurrence		
No	46	66.7
Yes	23	33.3
Follow‐up duration (mo)		
Mean (SD): 42.5 (35.2)		
Median (range): 29.0 (3.2‐145.1)		
Total area of leukoplakia (cm^2^)		
Mean (SD): 2.3 (1.9)		
Median (range): 1.8 (0.1‐10.6)		
Malignant transformation		
No	61	88.4
Yes	8	11.6

a One missing data in body mass index (n = 68).

b Thirteen out of 69 patients had multiple sites of oral leukoplakia, so the number of total lesions was 84.

The topographic subsites, pathology, and treatment outcomes of every OL lesion are demonstrated in Table 2. Thirteen of 69 patients (18.8%) had multifocal disease; therefore, the total number of lesions in the 69 patients was 84. The number of homogeneous and nonhomogeneous type of OL were equally distributed, with 42 cases each. Speckled type of OL (31 cases) outnumbered the nodular (nine cases) and verruciform (two cases) types. The buccal (64.3%) and tongue (14.3%) mucosa were the two most commonly affected regions, followed by the retromolar (8.3%), gum (5.6%), labial (4.8%), hard palate (1.2%), and mouth floor (1.2%). The number of cases of pathologically squamous hyperplasia, mild dysplasia, moderate dysplasia, and severe dysplasia/carcinoma in situ (CIS) was 22, 39, 14, and 9, respectively. If we adopt the binary classification system,^22^ the high‐risk lesions (23 cases, including moderate dysplasia and severe dysplasia/CIS) were not as many as the low‐risk lesions (61 cases, including squamous hyperplasia and mild dysplasia) (Table 2). Three patients had two different sites of recurrence; therefore, the number of recurrence lesions was 26 while the number of patients with recurrence was 23. Most of the recurrence occurred on the buccal mucosa (21 lesions), while the other five lesions were on the tongue (three lesions), retromolar (one lesion), and gum (one lesion). Malignant transformation occurred in eight patients, mainly in the buccal (four cases) and tongue (two cases) mucosa; the other two cases were located on the retromolar and labial mucosa, respectively (Table 2).

**Table 2:** The topographic sites, pathology, and treatment outcomes of different subsites (n = 84) of oral leukoplakia treated by carbon dioxide laser in the 69 elderly patients

	Buccal	Retromolar	Gum	Labial	Hard palate	Mouth floor	Tongue	Total
The number of lesions	54	7	5	4	1	1	12	84
Morphology								
Homogeneous	26	3	3	1	1	1	7	42
Non‐homogeneous	28	4	2	3	0	0	5	42
Speckled type	19	4	2	3	0	0	3	31
Nodular type	8	0	0	0	0	0	1	9
Verruciform type	1	0	0	0	0	0	1	2
Pathology								
Squamous hyperplasia	14	1	1	2	1	1	2	22
Mild dysplasia	25	5	3	0	0	0	6	39
Moderate dysplasia	10	0	1	1	0	0	2	14
Severe dysplasia/carcinoma in situ	5	1	0	1	0	0	2	9
Postoperative recurrence	21	1	1	0	0	0	3	26^a^
Malignant transformation	4	1	0	1	0	0	2	8

a There were two patients with more than one site of postoperative recurrence, so the number of recurrent lesions were 26 but the patients with postoperative recurrence were 23.

In the univariate analysis, morphology of OL, pathology, and the area of OL were the factors associated with postoperative recurrence (*P* < .05, Table 3). In terms of morphology, of 32 patients who had homogeneous OL, five developed recurrence; and of 37 with nonhomogeneous OL, 18 had recurrence (*P* = .005, OR 5.12, 95% CI 1.62‐16.18). Pathological results were categorized into four groups: squamous hyperplasia, mild dysplasia, moderate dysplasia, and severe dysplasia/CIS. Postoperative recurrence occurred in 2 out of 15 squamous hyperplasia, 10 out of 34 mild dysplasia, 5 out of 12 moderate dysplasia, and 6 out of 8 severe dysplasia/CIS (*P* = .02). The average area of OL without recurrence was 1.7 ± 1.3 cm^2^; those for OL with recurrence was 3.61 ± 2.34 cm^2^ (*P* < .001, OR 1.91, 95% CI 1.32‐2.76, Table 3). Other factors, including gender, age, body mass index, history of head and neck cancer, history of radiation therapy, cigarette smoking, alcohol drinking, betel quid chewing, candida infection, diabetes, taking metformin, multifocal disease, and subsites of OL were not significantly associated with postoperative recurrence. All the significant factors in the univariate analysis were included in the calculation mode of multivariate logistic regression analysis, which was adjusted for age and sex. Pathology and the total area of OL were the two independent predictive prognostic factor related to postoperative recurrence in the elderly patients with OL (*P* < .05, Table 4).

**Table 3:** Univariate analysis of the factors related to postoperative recurrence in the elderly patients who underwent surgical excision for oral leukoplakia

	Case no. (n = 69)	Postoperative recurrence		Odds ratio	95% CI	*P* value
		No (n = 46)	Yes (n = 23)			
Gender						.77
Female	16	10	6	1.0		
Male	53	36	17	0.79	.025‐2.52	
Age	69	71.8 ± 5.3	70.2 ± 3.9	1.60	0.83‐1.04	.20
Body mass index (kg/m^2^)^a^	69	24.3 ± 3.1	24.5 ± 3.5	1.01	0.87‐1.19	.86
History of head and neck cancer						.59
No	46	32	14	1.0		
Yes	23	14	9	1.47	0.52‐4.19	
History of radiation therapy						.16
No	58	41	17	1.0		
Yes	11	5	6	2.89	0.78‐10.78	
Cigarette smoking						.76
Non‐smoker	28	20	8	1.0		
Ex‐smoker	22	15	7	1.17	0.35‐3.93	
Current smoker	19	11	8	1.82	0.53‐6.19	
Alcohol drinking						.52
Non‐drinker	53	35	18	1.0		
Ex‐drinker	10	7	3	0.83	0.19‐3.61	
Current drinker	6	4	2	0.97	0.16‐5.82	
Betel quid chewing						.09
Non‐chewer	51	37	14	1.0		
Ex‐chewer	18	9	9	2.64	0.87‐8.02	
Current chewer	0	0	0	—	—	
Candida infection						.68
No	62	42	20	1.0		
Yes	7	4	3	1.57	0.32‐7.71	
Diabetes mellitus						1.0
No	52	35	17	1.0		
Yes	17	11	6	1.12	0.35‐3.55	
Metformin taken						.31
No	58	37	21	1.0		
Yes	11	9	2	0.39	0.077‐1.98	
Multifocal disease						.75
No (single lesion)	56	38	18	1.0		
Yes (multiple lesions)	13	8	5	1.32	0.38‐4.61	
Locations of leukoplakia^b^						.82
Buccal and other keratinized mucosa	71	48	23	1.0		
Tongue and floor of the mouth	13	10	3	0.88	0.35‐2.17	
Morphology						.005
Homogeneous	32	27	5	1.0		
Non‐homogeneous	37	19	18	5.12	1.62–16.18	
Pathology						.02
Squamous hyperplasia	15	13	2	1.0		
Mild dysplasia	34	24	10	2.71	0.51‐14.27	
Moderate dysplasia	12	7	5	4.64	0.71‐30.42	
Severe dysplasia/Carcinoma in situ	8	2	6	19.50	2.2‐173.49	
Total area of leukoplakia (cm^2^)	69	1.7 ± 1.3	3.6 ± 2.3	1.91	1.32–2.76	<.001

a One missing data in the group of patients with postoperative recurrence (n = 22).

b Thirteen out of 69 patients had multiple foci of oral leukoplakia, so the number of total lesions was 84.

**Table 4:** Multivariate logistic regression analysis of factors associated with recurrence of oral leukoplakia in the elderly patients

Variables	β	SE (β)	Odds ratio (95% CI)	*P* value
Morphology	0.87	0.76	2.4 (0.54‐10.52)	.25
Pathology	0.92	0.42	2.52 (1.11–5.74)	.028
Total area of leukoplakia (cm^2^)	0.74	0.23	2.1 (1.33‐3.29)	.0013

*Note*: Adjusted for age and sex.

**Figure 2:**
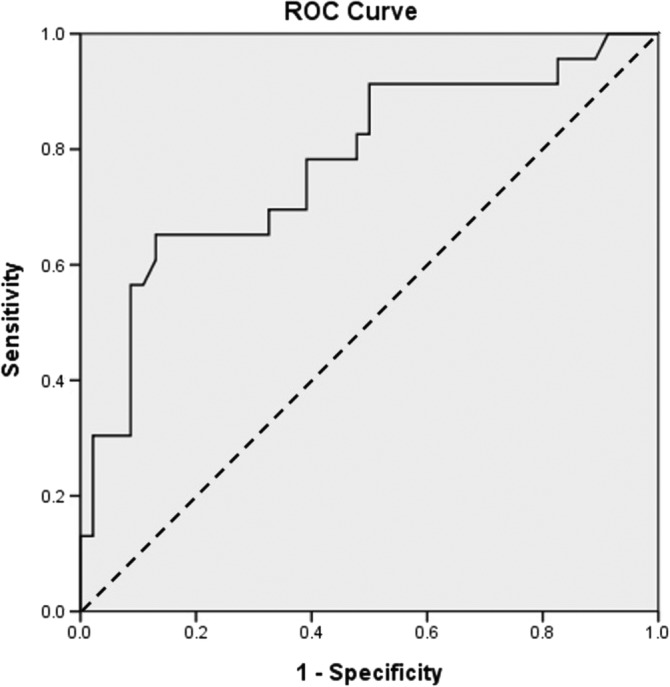
The receiver operating characteristic (ROC) curve analysis was used to predict postoperative recurrence. Each point on the ROC corresponds to a value on the area of oral leukoplakia. The area under the ROC curve (AUC) is 0.775. The straight dashed line represents the ROC curve expected by chance only

The mean area of the postoperative recurrent lesions (3.61 ± 2.34 cm^2^) was significantly larger than that of nonrecurrent lesions (1.70 ± 1.30 cm^2^). The cut‐off point of the area of 2.95 cm^2^ was calculated by the Youden index, and showed the best predictive value for postoperative recurrence (sensitivity = 0.65, specificity = 0.87); the area under the curve was 0.775 (Figure 2). The odds of postoperative recurrence was increased by 12.5 times (OR = 12.5, 95% CI 3.71‐42.07, *P* < .001) when the area of the lesion exceeded 2.95 cm^2^.

## 5 DISCUSSION

Several factors are associated with the occurrence and malignant transformation of OL; among these, cigarette smoking, alcohol drinking, and betel quid chewing are commonly mentioned.^25^, ^26^, ^27^, ^28^, ^29^, ^30^ In this study, morphologically nonhomogeneous type, pathology, and large area of OL were significantly associated with the treatment outcomes in the univariate analysis. Furthermore, the pathological high‐risk dysplasia and total area of OL remained as the two independent prognostic factors for postoperative recurrence in the elderly patients.

OL is a clinical diagnosis, so the morphological outlooks of OL are assessed by clinicians. In the present study, morphology was a significant factor associated with postoperative recurrence in the univariate analysis. In Table 2, the homogeneous and nonhomogenous types were distributed equally with 42 lesions each. Among the nonhomogeneous OL, speckled type was the most commonly encountered, which outnumbered the verruciform and nodular types of nonhomogeneous OL. The same phenomenon was also observed in other two studies of OL in Taiwan.^17^, ^27^ It is apparent that higher degree of dysplastic changes were more linked to nonhomogeneous OL than homogeneous lesions. Similar findings were also shown in other studies.^16^, ^33^ Clinicians should be more proactive when managing nonhomogeneous OL, with which more treatment failures and higher grade of dysplasia are often accompanied.

Histopathological examination is essential for the diagnosis of OPMDs, but the pathology per se is not sufficient to establish the diagnosis. Combination of clinical morphological outlooks and pathological features is the gold standard for the diagnosis of OPMDs. Although the pathological diagnosis can be obtained by way of a biopsy or surgical excision, discrepancy in pathological results between a biopsy and total excision should be kept in mind. There are inevitable sampling errors in biopsies and it is reasonable to deduce that a biopsy may not always represent the pathological condition of a whole lesion. Holmstrup et al demonstrated that biopsies of oral premalignant lesions might not be reliable and that discrepancy in pathology between the biopsied samples and that of excision of the whole lesion do exist.^34^ In the present study, all the patients received laser excision whether an initial biopsy was performed or not. The pathology was recorded with the highest degree of seriousness. Pathology was a significant factor both in the univariate (*P* = .02, Table 3) and multivariate analyses (OR = 2.52, 95% CI 1.11‐5.74, *P* = .028, Table 4) and was also one of the two independent prognostic factors of postoperative recurrence. In our previous study, pathological dysplasia was an associated factor with postoperative recurrence in the univariate analysis but not an independent prognostic factor.^16^ Here, we used the World Health Organization (WHO) classification to categorize the grades of dysplasia as mild, moderate, and severe.^3^ If we adopt the binary classification system (no/questionable/mild—low risk; moderate or severe—implying high risk),^22^ the statistical analysis result was also significant for postoperative recurrence (OR = 3.77, 95% CI 1.26‐11.27, *P* = .024, data not shown). Higher degree of dysplasia was associated with a higher postoperative recurrence rate. Currently it is not possible to determine the possibility of postoperative recurrence by the morphology or pathology. Genetically altered epithelial cells by routine histologic examination were even found in areas with normal histology,^34^ which may account for the potential inadequacy of the laser extirpation and the lack of clinical morphological correlation. Hence, identification of predictive molecular biomarkers related to the genetic and phenotypic alterations is required.^35^

The area of OL was another statistically significant factor both in the univariate and multivariate analyses (Tables 3 and 4). In a retrospective study of OL in 167 patients, several possible prognostic factors were identified, including age and size of the resected lesions. Lesion sizes were between 1 and 2 cm; the odds and >2 cm revealed ratio of disease‐free status as 1.76 and 2.18 times higher odds of disease‐free status, respectively, than lesions those less than <1 cm; if the area of the lesion was larger than 2.0 cm, the odds ratio was 2.18.^14^ Few studies addressed the effect of size of lesions on the postoperative recurrence of OL. In our study, lesions larger than 2.95 cm^2^ (Figure 2) showed a 12.5‐fold higher odds of postoperative recurrence (OR = 12.5, 95% CI 3.71‐42.07, *P* < .001). The larger the area is, the greater the possibility of recurrence will be. Although this might have been the trend in this study; however, a larger sample size is needed to further delineate the role of this factor.

Regarding surgical treatment as a tool to reduce malignant transformation of OL, it was reported as being effective in some studies,^36^, ^37^, ^38^, ^39^ but others had conflicting results.^13^, ^30^, ^40^, ^41^, ^42^, ^43^, ^44^ A report in the Cochrane Database of Systematic Review also indicated that there was no randomized clinical trial that has included a no treatment or placebo comparison group in investigating the role of surgical treatment for OL.^45^ The role of radical surgery for oral cancers has been established. The same concept more or less can be applied for the excision of OL. Although surgical excision cannot absolutely eliminate the possibility of malignant transformation (primary prevention), finding at histopathology from the excised specimens can provide an invaluable reference for further management (secondary prevention).^30^ Our previous work has shown that postoperative recurrence of dysplastic OL may be associated with malignant transformation.^17^ Clinicians should therefore be aware of treatment failure as a consequence of postoperative recurrence.

The habit of betel quid chewing is prevalent mostly in some local areas such as India and Taiwan.^26^ In the present study, we found that 18 out of the 69 patients had the habit of betel quid chewing but none were current chewers (Table 1). No statistical significance was found between the oral habits and postoperative recurrence in the univariate analysis (Table 4). Comparing the five published studies of OL in Taiwan,^17^, ^26^, ^27^, ^31^, ^32^ the proportion of patients who had never had the habits of drinking, smoking, and betel use in the present study was significantly lower (*P* < .001, Table 5), which implied that the pattern of oral habits of the elderly patients with OL may be different from the general population and its role needs more investigation.

**Table 5:** Comparison of oral habits in studies on oral leukoplakia conducted in Taiwan, a betel quid chewing endemic region

Author(s)	Location	Date	Case no. (n)	Age	Male/female	Ever alcohol drinking (%)^a^	Ever cigarette smoking (%)^a^	Ever betel quid chewing (%)^a^
Lee et al^31^	Taiwan	2003	125	47.9 ± 11.8	118/7	42.4% (53/125)	84.8% (106/125)	77.6% (97/125)
Shiu et al^26^	Taiwan	2004	164	46 ± 14	146/18	17.07% (28/164)	76.22% (125/164)	60.37% (99/164)
Lee et al^27^	Taiwan	2006	1046	50.0 ± 12.1	956/90	35.37% (370/1064)	80.78% (845/1064)	75.9% (794/1064)
Yang et al^17^	Taiwan	2010	114	49.7 ± 12.2	90/24	31.58% (36/114)	76.32% (87/114)	57.89% (66/124)
Chuang et al^32^	Taiwan	2018	5142	47	5142/0^b^	62.8% (3231/5142)	94.1% (4838/5142)	81.8% (4205/5142)
Present study	Taiwan		69	71.22 ± 4.89 (all >65)^c^	53/16	23.19% (16/69)^c^	59.42% (41/69)^c^	26.09% (18/69)^c^

a Ex‐users and current users were both included.

b Female patients were excluded in the study.

c *P* < .001, statistical comparison between the present study and the five published reports.

As for OL, the group of elderly patients seems to have its own unique characteristics. Reviewing the literature, few studies addressed the issue of OL in the elderly patients and this is the first cohort study describing the clinical characteristics and evaluating the treatment outcomes in elderly patients with OL. The results may serve as a preliminary reference for clinicians dealing with OL in the elderly.

There are some limitations in this study. First, there were few variables with missing data giving rise to an insufficient data collection and a high attrition rate due to the retrospective nature of the study. Secondly, the sample size was small. The proportion of elderly patients with OL is small compared with general population. It is difficult to collect a lot of patients in a single institution. In other words, multicenter, prospective, and randomized controlled studies are warranted to further investigate the characteristics and treatment outcomes of OL in the elderly patients.

## 6 CONCLUSIONS

Morphologically nonhomogeneous type, pathology, and large area of OL were significantly associated with the treatment outcomes in the univariate analysis. The pathological high‐risk dysplasia and total area of OL were the two independent prognostic factors of postoperative recurrence in the elderly patients. A more aggressive treatment for high‐risk dysplasia or lesions with a total area of more than 2.95 cm^2^ is suggested.

## CONFLICT OF INTERESTS

The authors declare no conflicts of interest.
